# Boosting and lassoing new prostate cancer SNP risk factors and their connection to selenium

**DOI:** 10.1038/s41598-021-97412-2

**Published:** 2021-09-09

**Authors:** David E. Booth, Venugopal Gopalakrishna-Remani, Matthew L. Cooper, Fiona R. Green, Margaret P. Rayman

**Affiliations:** 1 M&IS Department, Kent State University, 595 Martinique Circle, Kent, OH 44242 USA; 2 Department of Management, University of Texas-Tyler, Tyler, TX 75799 USA; 3 Department of Internal Medicine, Washington University School of Medicine, St. Louis, MO 63110 USA; 4 Division of Cardiovascular Sciences, School of Medical Sciences, Faculty of Biology, Medicine and Health, University of Manchester, Manchester, UK; 5 Department of Nutritional Sciences, University of Surrey, Guildford, GU27XH UK

**Keywords:** Cancer, Genetics, Biomarkers, Oncology, Risk factors

## Abstract

We begin by arguing that the often used algorithm for the discovery and use of disease risk factors, stepwise logistic regression, is unstable. We then argue that there are other algorithms available that are much more stable and reliable (e.g. the lasso and gradient boosting). We then propose a protocol for the discovery and use of risk factors using lasso or boosting variable selection. We then illustrate the use of the protocol with a set of prostate cancer data and show that it recovers known risk factors. Finally, we use the protocol to identify new and important SNP based risk factors for prostate cancer and further seek evidence for or against the hypothesis of an anticancer function for Selenium in prostate cancer. We find that the anticancer effect may depend on the SNP-SNP interaction and, in particular, which alleles are present.

## Introduction

In the present paper we introduce two newer variable selection method, the lasso and gradient boosting which we argue are large improvements to the often presently used methods^1^. We discuss the advantages of these newer methods and show how they successfully find new, as well as known, risk factors for prostate cancer. We then discuss what this means in the search for new anti-cancer drugs in the case of prostate cancer.

As Austin and Tu^1^ remark, researchers as well as physicians are often interested in determining the independent predictors of a disease state. These predictors, often called risk factors, are important in disease diagnosis, prognosis and general patient management as the attending physician tries to optimize patient care. In addition, knowledge of these risk factors help researchers evaluate new treatment modalities and therapies as well as help make comparisons across different hospitals^1^. Because risk factors are so important in patient care it behooves us to do the best job possible in the discovery and use of disease risk factors. Because new statistical methods^2–9^ have been and are being developed^8^, it is important for risk factor researchers to be aware of these new methods and to adjust their discovery and use of risk factor protocols as is necessary. In this paper, we argue that now is such a time. For a number of years in risk factor research a method of automatic variable selection called stepwise regression and its variants forward selection and backward elimination^10^ (chapter 9) have been used even as new methods have become available (see^11–17^ and many others). The last three cited are risk factor studies. We do not argue for a change of protocols in risk factor discovery and use simply because newer methods are available. As literature shows^1^ the older methods are often untenible and the newer methods are much less so. In particular, in a simulation study of stepwise methods, Austin and Tu^1^ found that 1,000 runs of backward elimination on the same data set produced 940 different “optimal” models. However, in our opinion, a bit more needs to be said about stepwise regressions and other similar variable selection approaches. As we just remarked it is possible to have a selection process to produce optimal models by these older approaches. Recall by optimal we mean that no better predictive solutions exist. In theory then we could find an optimal model by these methods. The problem is the identification of such a model because we may have many candidates. Thus as far as we know the only way to know if it is optimal is to test each candidate individually which is very labor intensive. As we discuss in the next section of the paper, the oracle property of adaptive lasso regression requires less laborious methods, Thus we recommend the use of adaptive lasso to solve our selection problem (i.e. the identification of disease risk factors) by this method. In addition in our recommended protocol we recommend use of a gradient boosting algorithm to verify our adaptive lasso solution. The disadvantage of gradient boosting as an overall solution is that it only identifies the salient risk factors and does not provide actual prediction equation to use to compute the actual risk itself as is needed to be certain these new risk factors have a realistic effect in changing the actual salient risks involved.

While a comparison between stepwise regression and lasso/gradient boosting was beyond the scope of the present work, the aim of which was to identify SNP based risk factors for prostate cancer. Austin and Tu^1^ have previously established the instability (in the sense that so many candidate models were produced by stepwise regression) it is practically too labor intensive for use in routine risk factor studies.

We point out that the overall purpose of this paper is twofold. First, we wish to introduce two methods of statistical variable selection and show how they can be used to identify disease risk factors, especially in the case of prostate cancer. Second, we wish to investigate these several new SNP risk factors for prostate cancer and using these risk factors see what we can discover more about the effect of selenium on prostate cancers. Our paper makes the following points:

1. We summarize some of the studies that show that stepwise regression and its variants, as now used more often than they should be in risk factor studies, are unreliable and in fact may cause some of the irreproducibility of life sciences research as discussed by^18^ as we shall discuss later.
2. We then argue on the basis of current research that there are methods available that are considerably more reliable.
3. We then propose a modern statistical protocol for the discovery and use of risk factors when using logistic regression as is commonly done.
4. We illustrate the use of the protocol developed in 3 using a set of prostate cancer data^19^.
5. We report the finding of new and important prostate cancer risk factors using the modern procedures. These new risk factors are important because they increase the possibility of an explanation for the potential anticancer effects of Selenium^19,20^ in prostate cancer. This is the reason for studying the SEPP1 and SOD2 genes. Cooper et al.^19^ discussed the possibly of such a mechanism. We quote from^19^. “Selenium may affect prostate cancer risk via its plasma carrier selenoprotein P which shows dramatically reduced expression in prostate cancer tumors and cell lines. The selenoprotein P (SEPP1)Ala234 single nucleotide polymorphism (SNP) allele is associated with lower plasma selenoprotein Pin men, reducing the concentration/activity of other antioxidant selenoproteins. Selenium status also modifies the effect of the mitochondrial superoxide dismutase (SOD2) SNP Ala16Val on prostate cancer risk.” This is a continuation of the earlier study^19^ which “investigated the relationship of these SNPs with prostate cancer risk”.

We further note that nothing in the way of statistical methods is new in this paper. What is new is the introduction of a clear protocol to identify and use disease risk factors that involve much less problematic methods than stepwise regression. We then use the proposed protocol to identify a known prostate cancer risk factor and then discover new and important prostate cancer risk factors and finally see what conclusions can be drawn about the relationship between selenium and prostate cancer. In particular we propose a new hypothesis that may explain the contradictory results on the relationship of Selenium and prostate cancer.

### What then should replace these automatic variable selection methods?

From the references in “Introduction” section, we see that the shrinkage methods have done well when compared to the current stepwise and all subsets methods and thus we follow the suggestion of Steyerburg et al.^4^ and look at shrinkage methods. The question then becomes what shrinkage method might we choose as the next variable selection method? We are impressed by the work of Ayers and Cordell^2^ in this regard. First, we note that shrinkage estimators are also called penalized estimators. In particular the lasso^7^ as defined by Zou^21^ can be considered. We note that the factor lambda is said to be the penalty because it weights one term in the definition^21^ more than the other. This is because the weight for one term is lambda and for the second term is 1-lambda. As lambda changes from 0 to 1 the weights are adjusted accordingly. This adjustment can be optimized for a particular data set by using techniques of mathematical optimization. The adjustment and method are discussed with respect to the figures and protocol later in the presentation. We mention there that different variable types require different optimization methods and provide more details and references. This fact leads to calling this approach penalization by some authors as discussed in the following paragraph.

Now Ayers and Cordell^2^ studied “the performance of penalizations in selecting SNPs as predictors in genetic association studies”, where SNP stands for single nucleotide polymorphism. Their conclusion is: “Results show that penalized methods outperform single marker analysis, with the main difference being that penalized methods allow the simultaneous inclusion of a number of markers, and generally do not allow correlated variables to enter the model in which most of the identified explanatory markers are accounted for”, as shown by Tibshirani^7^. In addition, lasso prevents overfitting the model^9^, p 304. At this point, penalty estimators (i.e. shrinkage) look very attractive in risk factor type studies^9^ (chapter 16.), especially given the relationship between lasso and boosting^9^, p. 320.

Another paper^21^ helps us make our final decision. Zou^21^ considers a procedure called adaptive lasso in which different values of the parameter λ are allowed for each of the regression coefficients. Furthermore, Zou shows that an adaptive lasso procedure is an oracle procedure such that β(Ϩ) (asymptotically) has the following properties.

- It identifies the right subset model and
- It has the optimal estimated rate.

Zou then extends these results to the adaptive lasso for logistic regression. Wang and Lang^22^ developed an approximate adaptive lasso (i.e. a different λ for each β is allowed) by least squares approximation for many types of regression. Boos^23^ shows how easy it is to implement this software in the statistical language R for logistic regression. Thus, we choose to use the least squares approximation to their adaptive lasso logistic regression in the next section. We note here that a special variant of lasso, group lasso^24^ is needed for categorical predictor variables.

In the next section, we propose and discuss a protocol for the discovery and use of risk factors in logistic regression models. In the following section we illustrate the use of the protocol using the data of Cooper et al.^19^ to look at some risk factors for prostate cancer. We will show that currently known risk factors can be identified as well as new risk factors discovered using these methods.

In addition, a second new method of variable selection called gradient boosting has been developed^25–27^, Chapter 8^9,28^, (Chapter 17.). This method has some of the same advantages as lasso and we add it to the protocol and test it as well. The boosting method makes use of regression trees. A readable introduction can be found in^29^. The main purpose of the boosting algorithm is to further confirm the lasso results.

## Materials and methods

### A suggested protocol for using logistic type regression to discover and use disease risk factors

Our suggested protocol is shown below. We discuss the protocol in this section and illustrate its use with prostate cancer risk factors in the following section. This protocol uses the R statistical language. R was chosen because of its power and the fact that all of the required algorithms are available in R. See^23^ for their internet URLs.

#### Protocol for use with risk factors

**Figure 1:**
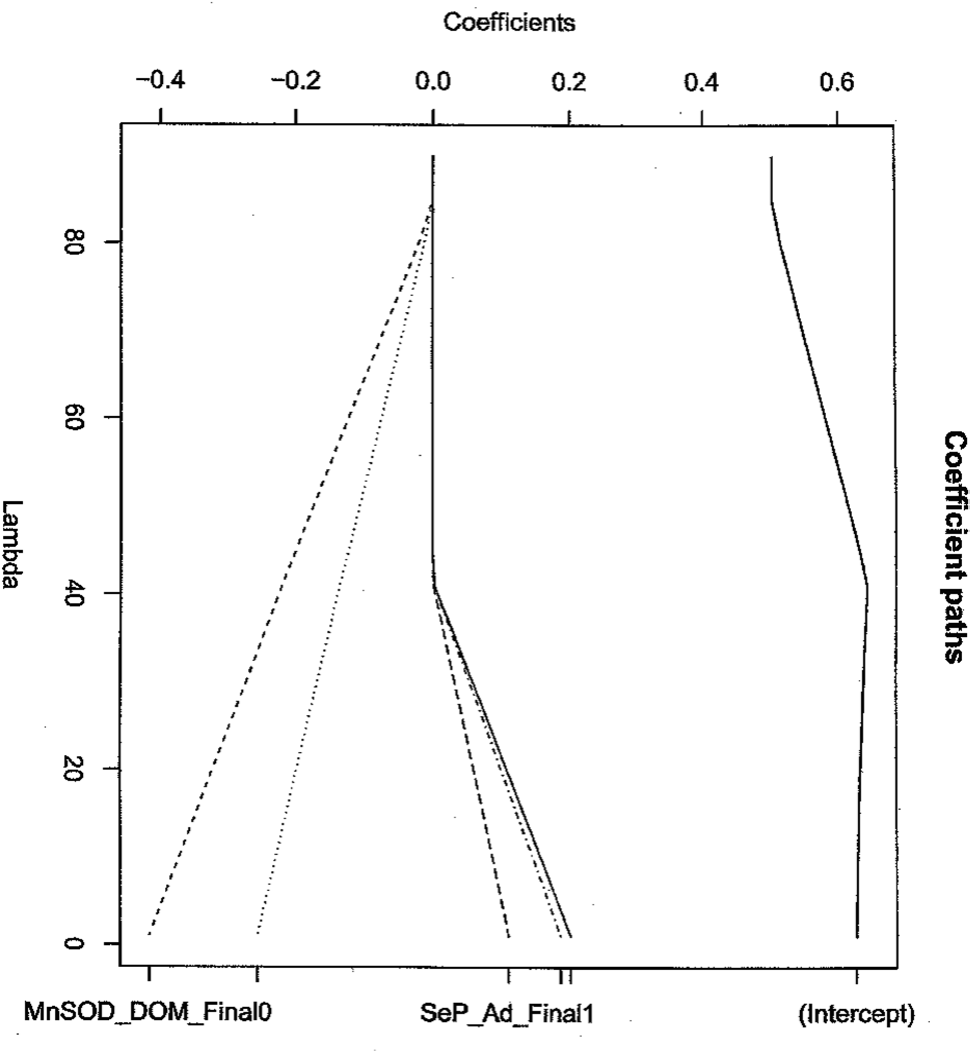
The Group Lasso Coefficient plot for the logistic regression. Containing MnSOD_DOM_FINAL and SeP_Ad_Final. We note that for lambda = λopt none of the paths shrink to zero suggesting that a SNP-SNP interaction, as reported in^19^ exists.

1. Ready data for analysis.
2. Input to R.
3. Regress a suitable dependent variable ((say) 0—Control, 1—Has disease) on X (a potential risk factor) as described by Harrell^30^ (Chapter 10) for logistic type regression.
4. Select a set of potential risk factors. If an X variable is continuous, we suggest use of the Bianco-Yohai (robust (outlier resistant), see^31^) estimator and further suggest putting outliers, sometimes called leverage points, aside for further analysis as they may give rise to extra information^31^. This step can help to lessen the effects of anomalous data points
5. Now build a full risk factor prediction model as described by Shmueli^32^.
6. Use potential risk factors (Xs) to form a full model with the appropriate dependent variable (as in 3).
7. If any variables are continuous repeat 4 using the entire potential full prediction model.
8. With any outliers set aside for further study, regress the dependent variable on the logistic regression type full model using the adaptive lasso method, least squares approximation, as described by Boos^23^.
9. Using a Bayesian Information Criterion (BIC) or alternatively an Akaike Information Criterion (AIC), select variables without zero lasso regression coefficients to be predictors in a risk factor based reduced model^23^. If categorical risk factors are present, use group lasso regression^24^. Use graphs like Fig. 1 in^24^ to identify the zero lasso regression coefficients that may exist for the categorical variables.
10. Repeat Step 8 for gradient boosting as described by Kendziorski^26^ or Ho^33^.
11. Validate the reduced model, with the similar validation of the full model of step 6, if there is any doubt about variables discarded from the full model, using bootstrap cross validation or tenfold cross validation^30^ and then check the usual model diagnostics^34^ for either lasso or boosting or both.
12. Predict with the reduced model containing the appropriate risk factors as described in Harrell^30^, Chapter 11 and Ryan^35^, Chapter 9.

Notes to the protocol.

A. We note that for the genome wide case of predictors one should refer to^36^ and^37^.
B. All logistic regression assumptions should be checked and satisfied as in Pregibon^27^.

## Results

### The prostate cancer case

This example is taken from Cooper et al.^19^ where the data (including all sample sizes) and biological system are described. Also see the data description in the “Appendix”. The data set used in this paper is a subset of the Cooper et al. data set with all observations containing missing values of model variables removed. Further we note that all potential predictor variables are categorical, so no imputation was performed. The coding assignments and the variable definitions are given in the “Appendix”. The simple and multiple logistic regressions are carried out as described in^30^. Robust logistic regressions, when needed, are carried out as described in^31^. Variable selection is carried out using the adaptive lasso^21^ with the least squares approximation of Wang and Leng^22^ for continuous independent variables and by group lasso^24^ for categorical independent variables. Gradient boosting is carried out using R Package gbm^25^ as described by^26,28,33^. All computations are carried out using the R statistical language. The R functions for variable selection (adaptive lasso and group lasso) along with the papers are available from Boos^23^, and used as described there. The use of the group lasso R function is covered in R help for packages grplasso and grpreg. The data sets and R programs are available from the authors (DEB). Further the R code can be found at the URLs given in^23^ and the data can be found on the internet as indicated in the “Appendix”. The variables studied as potential risk factors are listed in the X column of Table 1. The dependent variable is current status. The goal of the research is to see if our results support a possible mechanism for Selenium’s anticancer function in prostate cancer as suggested in^19^.

**Table 1:** Simple logistic regression results dependent variable CURRENTSTATUS intercepts are not listed.

X	Coeff	SE	P
X_STRATUM	− .055132	.005646	< 2 × 10^−16^
**MnSOD_AD_Final**			
0	− 0.4334	.1241	0.000477
1	− 0.2478	.1157	0.032196
2	− 0.3140	.1233	0.010879
**SeP_Ad_Final**			
0	0.21219	0.10309	0.039557
1	0.12890	0.10754	0.230675
2	0.23484	0.15797	0.137117
**MnSOD_DOM_Final**			
0	0.4334	0.1241	0.000477
1	0.2704	0.1126	0.016369
**SeP_DOM_Final**			
0	0.21219	0.10309	0.039557
1	0.14445	0.10568	0.171679
**Smoke_ever**			
0	− .00339	.08161	0.967
1	− .03791	.07016	0.589
**Alco_ever 0**			
0	− 0.428943	0.142425	0.0026
1	0.002951	0.062317	0.9622
FAMHIST	0.84619	0.09497	< 2 × 10^−16^

**Figure 2:**
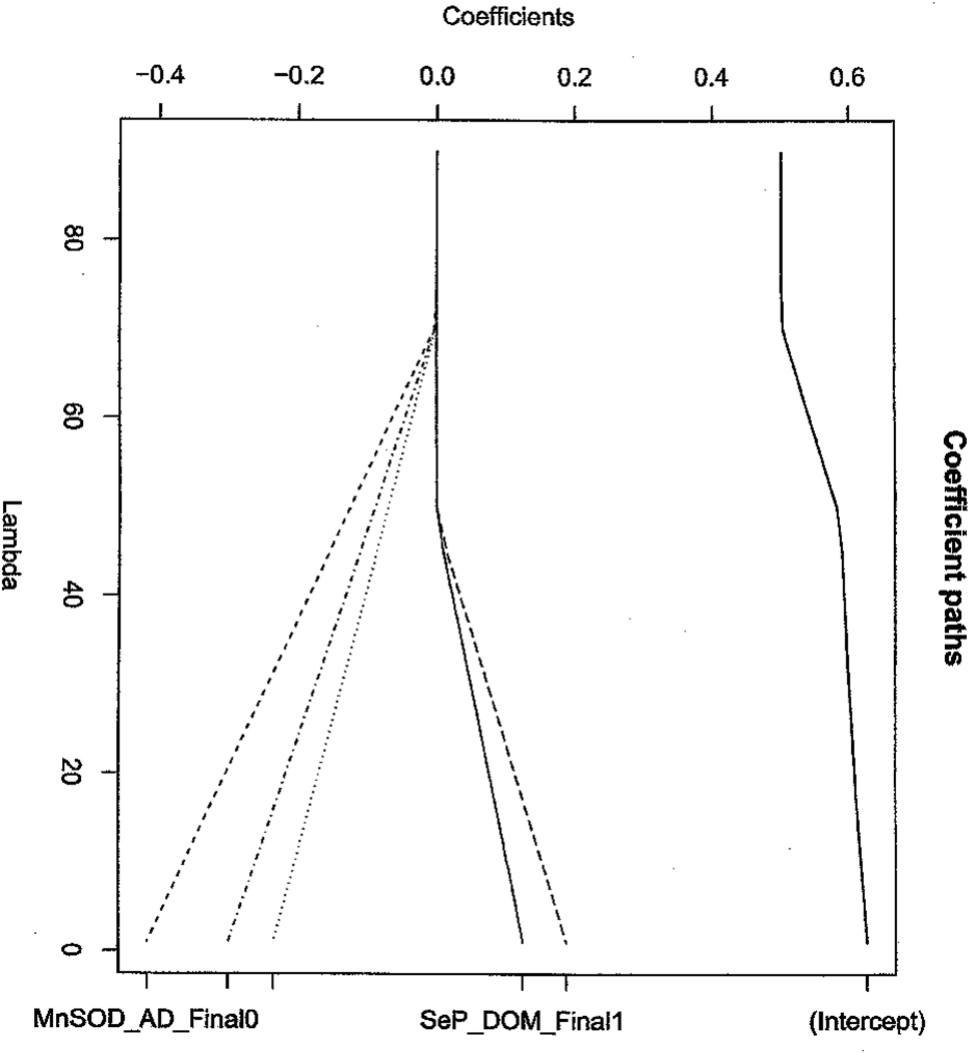
Group lasso Coefficient Plot for Model Containing MnSOD_AD_Final and SeP_DOM_Final.

We now follow the protocol and explain each step in detail. We begin by considering the one predictor logistic regressions in Table 1. First note that all potential risk factors in this data set are categorical (factors) so we do not have to consider the Bianco-Yohai^38^ estimator of protocol Step 4 for this data. We note that this is often not the case. Cooper et al.^19^ hypothesize a SNP-SNP interaction as a risk factor for prostate cancer where SNP denotes a single nucleotide polymorphism. Recall point 5 of “What then should replace these automatic variable selection methods?” section. We now test this hypothesis and attempt to answer the question is there such an interaction? In order to answer this question, we first note that the answer is not completely contained in Table 1. Second, we recall that we have a gene–gene interaction of two genes if both affect the final phenotype of the individual together. To be specific, we now consider the two genes representing the relevant alleles of the SEPP1 and SOD2 genes, the genes involved in the potential mechanism for selenium anticancer properties. If there is a gene–gene interaction, we must see the following statistically. The relevant alleles of the SEPP1 and SOD2 genes must be selected to be in a reasonable prediction equation for the disease state by the appropriate lasso or boosting algorithm (see Figs. 1, 2, Tables 3, 4). The appropriate lasso algorithm here is the group lasso for logistic regression because the predictor variables are categorical. We now note that in our data set we have four candidate predictor variables from which to search for our gene–gene interaction MnSOD_DOM_Final, SeP_Ad_Final, MnSOD_AD_Final and SeP_DOM_Final. Either observation of the Variable Values or a simple trial shows that we cannot include all four variables in the model at once because they are pairwise collinear. Hence, we have to separate the variables into the two cases, the models of Figs. 1 and 2. We also note that lasso generally does not allow correlated variables to enter the model^2,7,9^ as well as prevents overfitting^7,9^.

We now begin our search using lasso with the model of Fig. 1. This gives us a candidate for an interaction. We then perform the group lasso analysis of Fig. 1. Here we must determine if the relevant alleles are included in the group lasso selected prediction equation. Roughly this is the case if the lasso regression coefficients are essentially not zero at the end of the algorithm’s execution as shown on the coefficient path plot of Fig. 1. By looking at equation (2.2) of^24^ we see that 0 ≤ λ < ∞ hence as λ → ∞, s_λ_(β) → 0 and thus β_i_ → 0 but not uniformly. Hence the question is what value of λ do we choose to determine if the coefficients are close enough to zero to discard that term from the model as a zero coefficient. Based on Table 2 where we compute the optimal λ to use we choose λ = 1.428 to be the cutoff point. Hence, we can now apply the condition of the previous paragraph. We now check Fig. 1 to see which if any of these candidate alleles are selected for the group lasso prediction equation which was our criterion. We now examine the Fig. 1 plot at λopt = 1.428. We note that at this λ none of the candidate alleles have coefficients of zero. Hence using our criterion, we can summarize as follows:

1. We need Fig. 1 selection to show interaction. SeP_Ad_Final0 was Ala/Ala so this is one allele that qualifies. Similarly, for SeP_Ad_Final1 and 2 which are Ala/Thr and Thr/Thr respectively.
2. Both MnSOD_DOM_Final0 and MnSOD_DOM_Final1 (i.e. Ala/Ala and + /Ala) satisfy so this shows that for MnSOD the result is + /Ala. Hence the identified interaction alleles are

**Table 2:** Optimal λs computed from R packages grplasso and grpreg for indicated models.

Predictors in model	λmin	λmax	λopt
MnSOD_AD_Final SeP_DOM_Final	.009	70.55	.635
MnSOD_DOM_Final SeP_Ad_Final	.017	83.99	1.428

λmin computed by package grpreg using a Bayesian Information Criterion λmax was computed by package grplasso.

**Table:** 

Gene name	Value
SEPP1	Ala/Ala
SOD2	+ /Ala

which agrees with the Cooper et al.^19^ finding on a gene–gene interaction risk factor.

### New risk factors

Similarly, we have from SeP_Ad_Final 1 and 2

**Table:** 

Gene name	Value
Ala/Thr	+ /Ala
Thr/Thr	+ /Ala

which are also risk factors.

We now repeat this analysis for the model which contains the other possible candidate alleles. By our criterion for gene–gene interaction we need β_i_ ≠ 0 for λopt = 0.635, from observing Table 2. Now by observing Fig. 2 we see that for MnSod_Ad Final the 0, 1 and 2 values meet the criteria while for SeP_DOM_Final only the 0 and 1 alleles do. By consulting the “Appendix”, we see that

- SeP_DOM_Final1 is Ala/Thr and Thr/Thr
- SeP_DOM_Final0 is Ala/Ala
- MnSOD_AD_Final0 is Val/Val
  - 1 is Val/Ala
  - 2 is Ala/Ala

Hence, we conclude that we have additional gene–gene interactions that are risk factors. Since one combination was identified using the first model. We now have

**Table:** 

SEPP1	SOD2
Ala/Ala	Val/Val
Ala/Ala	Val/Ala
+ /Thr	Val/Val
+ /Thr	Val/Ala

as risk factors. None of these have been reported in the prior literature as far as we can determine.

We can now make prediction equations using our now known risk factors which will give our predicted diagnosis of whether or not a patient is at risk for prostate cancer based on our variable values assuming that we use a new observation not one which is included in our current dataset. We note from Figs. 1 and 2 that some risk factor coefficients seem to be positive and some negative. We note that this could mean that some alleles lead to an antitumor effect while other alleles are tumorigenic. Thus we believe follow up of the current study is most important. This observation may have some bearing on why Selenium is sometimes reported as an anti-cancer compound, but not always. We recommend and use bootstrap cross validation to validate this equation and full details are included in^30^. As a final reminder, all of the other assumptions of logistic regression need to be checked each and every time such a model is used. The reader is referred to Pregibon^34^ for further details. These new risk factor results are particularly important since the SEPP1 gene product is in the same metabolic path as a tumor suppressor for prostate cancer^20^. This may help provide a mechanism for selenium’s possible anti-prostate cancer action^19^. We also notice in Figs. 1 and 2 the regression coefficient plots show both negative and positive values as the curve proceeds to convergence. This suggests that we have both positive and negative risk factors in this data and hence some risk factors suggest anticancer activity while others suggest positive tumorigenic cancer activity. This perhaps suggests that some combinations of these alleles are anticancer while others are cancer causing. If this is the case, then perhaps we have a start on explaining the complicated mechanism that seems to be in operation between Selenium and prostate cancer.

We now repeat the analysis using gradient boosting. The purpose of this analysis is to verify and confirm the lasso results. Please notice that in this paper each new result has been verified by at least two independent methods. That is the point of adding gradient boosting to demonstrate to the reader that our results are reproducible and solid. The results are shown in Tables 3 and 4. The results are identical to the lasso results in the sense that exactly the same risk factors are obtained.

**Table 3:** Boosting results Pkg gbm Ada Boost, corresponds to Fig. 1.

Variable	Relative influence
MnSOD_DOM_Final	68.96
SeP_Ad_Final	31.03

**Table 4:** Boosting results, same conditions as Table 3, corresponds to Fig. 2.

Variable	Relative influence
MnSOD_AD_Final	75.29
SeP_DOM_Final	24.70

## Discussion

### Limitations of the proposed protocol and future research

As much as we would like this to be the last word on the discovery and use of disease risk factors with logistic regression, it is not. We will mention a few possible limitations and our hope for some future work perhaps by us or others that we would like to see.

First, Ayers and Cordell^2^ mention a limitation of this suggestion, the fact that there is no known way to get confidence intervals and p-values for lasso estimates,i.e. the lasso regression coefficients. Fortunately, this is changing. There is a paper by Lockhart et al. entitled “A significance test for the lasso”^39^. While this is a complicated paper that doesn’t solve all problems a strong beachhead has been established. Unfortunately, this is not a test on individual lasso regression coefficients but rather an omnibus test.

Next, we discussed the advantages of adaptive lasso earlier (esp. the oracle property) but no algorithm currently exists to solve the adaptive group lasso problem in the case of logistic regression. We conjecture based on the results of the linear regression case extended to the logistic case that if we could extend adaptive lasso to the group lasso for logistic regression cases that the same desirable properties of adaptive lasso would hold, especially the oracle property.

Finally, the usual problems of outliers, etc., as always, raise their head. The Bianco-Yohai algorithm^38^ is a start. This type thinking has been extended to some penalized shrinkage regression methods, but not yet for logistic regression to our knowledge. We conclude that there is much work to be done and fully expect to see other papers like this one in the future and hopefully statistical practice can continue to evolve and even better solutions can be applied to these interesting and important problems.

### Selenium as an anti-prostate cancer compound

We have found additional risk factors involving the SNPs in SEPP1 and SOD2. This provides support for a possible anti-prostate cancer function for selenium in addition to those reported by Cooper et al.^19^, hence continuing to support a reported anticancer effect^40^ for selenium^19,20^. However as we noted above that the regression coefficient plots may suggest some of these combination are anticancer while some are tumorigenic. This observation may shed light on what seems to be a complicated relationship between Selenium and prostate cancer.

## Conclusion

We have attempted in this paper to bring up to date statistical thinking to the problem of the identification and use of disease risk factors, where stepwise regression is still too often used. Much remains to be done, but we hope that the ideas presented here will improve statistical practice in this very important area. In the process of bringing this thinking up to date, we have shown that we recover a currently known risk factor and identify new risk factors for prostate cancer which suggest the value of our approach. These new risk factor results are particularly important since the SEPP1 gene product has recently been shown to be in the same metabolic pathway as a tumor suppressor (Selenium binding Protein 1) for prostate cancer^20^. This further supports the possibility that selenium has anti-prostate cancer properties^19^ but may have tumorigenic properties as well with certain genotypes. This could explain why studies like SELECT have shown a positive cancer effect and yet other studies show an anti-cancer effect for selenium^20,41–43^. This suggests a complicated mechanism. Figures 1 and 2 suggest different signed regression coefficients for the different alleles. Again, this could suggest that different alleles in a codon could cause different effects induced by the resulting protein and hence different biological activity for the resulting protein. Our working hypothesis is that this effect is related to the SNP-SNP interaction. Perhaps differences such as these are causing reports of positive and negative selenium effects for anti-cancer activity. Only more research will shed light on this complicated area.

## Data Availability

This Data can be obtained on ResearchGate.net at David Eugene Booth’s account with https://doi.org/10.13140/RG.2.2.19989.86240.

## Appendix: Data set

Total number of observations is 4679.

This Data can be obtained on ResearchGate.net at David Eugene Booth’s account with https://doi.org/10.13140/RG.2.2.19989.86240.

**Table:**

INCLUSIONSTATUS	Cancer status at inclusion	0 = Control
		1 = Cancer
X_INCLUSIONAGE_YRS	Age	Age
CURRENTSTATUS	Updated cancer status	0 = Control
		1 = Cancer
T	T-stage	Staging 1 to 4
		− 1 = Control
		9 = No data
N	N-stage	0 = N −
		1 = N +
		− 1 = Control
		99 = No data
M	M-stage	0 = M −
		1 = M +
		− 1 = Control
		99 = No data
GLEASON	Gleason score	Staging 1 to 10
		− 1 = Control
		99 = No data
PSA	Prostate specific antigen	μg/ml
		− 1 = Data not available
		− 2 = Control
ADV	Advanced stage cancer in at least one of the above markers (TNM, Diff, Gleason, PSA) see below for how the cancers were classified	0 = Not aggressive
		1 = Aggressive
		− 1 = Control
		99 = No data
X_STRATUM	Stratification of data based on age and geographical location	
FAMHIST	Family history	0 = No
		1 = Yes
smoke_ever	Smoking	0 = Never
		1 = Ever
		99 = Data missing
alco_ever	Alcohol consumption	0 = Never
		1 = Ever
		99 = Data missing
X_BMI	Body mass index	− 1 = No data
		1 < , = BMI
MnSOD_AD_Final	SOD2 genotype	0 = Val/Val
		1 = Val/Ala
		2 = Ala/Ala
MnSOD_DOM_Final	SOD2 dominant model	0 = Val/Val
		1 = Val/Ala and Ala/Ala
SeP_Ad_Final	SePP1 genotype	0 = Ala/Ala
		1 = Ala/Thr
		2 = Thr/Thr
SeP_DOM_Final	SePP1 dominant model	0 = Ala/Ala
		1 = Ala/Thr and Thr/Thr
inclusion_age_banded	Age banded within 10 years	
Ad_control_100_final	Aggressive and control. All other cases excluded	0 = Control
		1 = Aggressive
Loc_control_100	Non-aggressive and control. All other cases excluded	0 = Control
		1 = Non aggressive

Cases were classified as either non-aggressive at diagnosis (tumor stage 1 and 2, Gleason score < 8, Differentiation G1-G2, NP/NX, MO/MX, PSA < 100 μg/L; NPC) or aggressive at diagnosis (tumor stage 3–4, Gleason score ≥ 8, Differentiation G3-G4, N + , M + , PSA ≥ 100 μg/L;APC).Total number of observations was 4679.

## Code availability

This example is taken from Cooper et al.^19^ where the data (including all sample sizes) and biological system are described. Also see the data description in the “Appendix”. The data set used in this paper is a subset of the Cooper et al. data set with all observations containing missing values of model variables removed. Further we note that all potential predictor variables are categorical, so no imputation was performed. The coding assignments and the variable definitions are given in the “Appendix”. The simple and multiple logistic regressions are carried out as described in^30^. Robust logistic regressions, when needed, are carried out as described in^31^. Variable selection is carried out using the adaptive lasso^21^ with the least squares approximation of Wang and Leng^22^ for continuous independent variables and by group lasso^24^ for categorical independent variables. Gradient boosting is carried out using R Package gbm^25^ as described by^26,28,33^. All computations are carried out using the R statistical language. The R functions for variable selection (adaptive lasso and group lasso) along with the papers are available from Boos^23^, and used as described there. The use of the group lasso R function is covered in R help for packages grplasso and grpreg. The data sets and R programs are available from the authors (DEB). Further the R code can be found at the URLs given in^23^ and the data can be found on the internet as indicated in the “Appendix”. The variables studied as potential risk factors are listed in the X column of Table 1. The dependent variable is current status. The goal of the research is to see if our results support a possible mechanism for Selenium’s anticancer function in prostate cancer as suggested in^19^.
